# Visible Pregnancy, Invisible HIV: How Social Norms Shape Adolescents Protection Choice in Kenya and Nigeria

**DOI:** 10.5334/aogh.5092

**Published:** 2026-01-12

**Authors:** Cassange Bitère, Raphael Joshua Ifenna, Hilawit Gebrehanna, Dorcas Nyasani Ombasa

**Affiliations:** 1 Senior Evaluation Consultant, Hillman Associates, LLC; Research Consultant, CARE USA, Atlanta, GA, United States of America; 2 Monitoring Evaluation Accountability and Learning Officer, Maiduguri, CARE Nigeria, Nigeria; 3 Direction – Behavior and Social Change, CARE USA, Atlanta, GA, United States of America; 4 Social Inclusion Lead, CARE Kenya, Nairobi, Kenya

**Keywords:** social norms, adolescent sexual health, HIV prevention, pregnancy stigma, contraceptive method, condoms, Kenya, Nigeria, sub-Saharan Africa

## Abstract

Adolescents in sub-Saharan Africa continue to face a high burden of HIV, with adolescent girls and young women disproportionately affected. Yet despite major investments in HIV education, testing, condoms, and biomedical prevention such as PrEP, reductions in new infections remain slow in countries like Kenya and Nigeria. This viewpoint argues that current prevention strategies overlook the social realities that shape adolescent decision-making. Drawing on Bicchieri’s Social Norms Theory and field experience, we highlight how visible sanctions tied to pregnancy, reputation, and respectability guide protection choices more than biomedical logic. For many adolescents, discreet hormonal contraception allows girls to avoid public shame, while boys often avoid condoms because being seen purchasing them signals sexual activity and attracts moral judgment. These gendered pressures reduce dual protection even when knowledge and access exist, and fragmented HIV and reproductive health services reinforce the divide. Health systems often deepen this pattern when adolescents feel judged or questioned by providers. Effective HIV prevention requires confronting these normative pressures and integrating youth-friendly reproductive and HIV services. Prevention becomes stronger when it aligns with how adolescents interpret risk, dignity, and responsibility within their social environments rather than focusing on biomedical solutions alone.

## Introduction: Persistent Inequity

Sub-Saharan Africa continues to carry the world’s highest HIV burden, with adolescent girls and young women (AGYW) disproportionately affected. An estimated 1.9 million (1.1–2.5 million) adolescent girls and young women aged 15–24 years and 1.2 million (840,000–1.6 million) adolescent boys and young men (ABYM) aged 15–24 years are living with HIV globally [1]. In Kenya, adolescents and young people aged 15–24 years account for a notable share of new HIV infections, with adolescent girls experiencing a much higher likelihood of HIV infection compared to their male peers [1]. Nigeria has one of the world’s largest adolescent populations, and although national HIV prevalence is relatively low at about 1.4%, young women aged 15–24 are more likely to be infected than their male peers [2, 3]. Despite major investments in prevention, the decline in new infections among AGYW and ABYM remains an issue.

While extensive investments have expanded HIV education, testing, condoms access, and biomedical prevention options such as Pre-Exposure Prophylaxis (PrEP), adolescents continue to assess risk through lenses that these biomedical approaches do not fully capture. For many young people, especially adolescent girls and boys, decisions about sex and protection are shaped less by medical risk and more by the social consequences attached to pregnancy, relationships, and reputation in their community. In Kenya, one qualitative study found that young women “don’t fear HIV; they fear pregnancy because everyone can see it” [4]. A similar dynamic appears in Nigeria, where the Gender Norms Learning Agenda (GNLA) report shows that unintended pregnancy carries visible social and economic consequences, including family shame, school disruption, and community judgment, while HIV remains deeply stigmatized and difficult to discuss openly [5]. This disconnect between biomedical framing and adolescents’ lived realities highlights a critical blind spot in current prevention strategies: social norms, not knowledge alone, shape how risk is perceived and acted upon.

These patterns are not isolated examples. Across Kenya and Nigeria, adolescents interpret risk through social meaning rather than biomedical logic. Drawing on field experiences in both countries and evidence from existing literature, this viewpoint examines how fear, stigma, and community expectations create a hierarchy of perceived dangers in which pregnancy carries visible social consequences, and HIV is seen as a distant or hidden threat. Understanding this hierarchy is a practical and programmatic necessity. Effective HIV prevention must begin with what adolescents truly fear and why.

The focus on Kenya and Nigeria is grounded in the authors’ direct engagement through CARE’s GNLA, which was a multi-country social norms diagnostic research that explored five sectors, including sexual and reproductive health (SRH), that are priority areas of outcome for adolescent girls and young women. Between 2023 and 2024, the GNLA conducted qualitative research with adolescents, parents, and service providers across various communities in both countries. This perspective combines insights gathered from that research with the authors’ shared experiences in implementing and supporting adolescent HIV and SRH programs in Kenya and Nigeria. These two countries provide valuable insights as they feature large adolescent populations along with ongoing gender disparities in HIV infection rates and intricate social expectations around sexuality, respectability, and the stigma surrounding pregnancy. As public and global health practitioners and researchers, we produce this work from our professional engagement in adolescent HIV and SRH programming in Kenya and Nigeria.

Additionally, we focused on peer-reviewed and grey literature articles on adolescent HIV prevention, sexual and reproductive health, and social norms in Kenya and Nigeria. The literature was examined alongside findings from the GNLA and the authors’ field experience to identify consistent patterns in how norms shape adolescents’ protection choices. The aim was to synthesize key insights rather than provide an exhaustive review.

These dynamics discussed in this article align with the Social Norms Theory, which explains how behaviors are shaped by shared expectations, anticipated sanctions, and the need to maintain social approval within one’s reference group [6]. The framework highlights how anticipated sanctions, the prospect of social rewards, and the visibility of certain behaviors determine whether individuals conform to or resist a norm. Applying this framework helps clarify why adolescents often prioritize avoiding visible social consequences, such as pregnancy-related stigma, over avoiding biomedical risks like HIV. When maintaining reputation, avoiding judgment, and adhering to community expectations carry greater social weight, decisions about condoms and contraception become guided by these normative pressures rather than clinical risk alone.

## The Paradox in Practice: Condoms Signal “Promiscuity,” Leading Adolescents to Prioritize Pregnancy Prevention Alone

Across Kenya and Nigeria, condoms carry a social meaning that extends beyond disease prevention. For many adolescent boys, buying or carrying condoms is perceived as public evidence of sexual activity and attracts moral judgment from shopkeepers, peers, and community members [5, 7]. The anticipated stigma associated with being seen obtaining condoms discourages open use. In this context, unmarried couples often avoid condoms not because they lack knowledge, but because condoms signal a violation of social expectations. On the other hand, married couples avoid condom use to prove fertility and marriage worthiness [8].

This dynamic then shifts responsibility for protection to girls. Pills and injectables are valued for their discretion and their ability to prevent the most socially visible consequence of sexual activity: pregnancy. However, these methods do not protect against HIV or other sexually transmitted infections. Studies in Kenya and Nigeria show that when pregnancy carries a greater social risk than HIV, dual protection becomes uncommon and condoms are routinely excluded from their method of choice, leading to infections [9, 10].

Knowledge and access alone are insufficient to change this pattern. Condom use is constrained not only by availability, but by the social meaning attached to being seen acquiring or carrying them. Unless prevention efforts address these social meanings and normalize dual protection for both partners, adolescents will continue to prioritize methods that preserve secrecy and social respectability rather than comprehensive protection.

## The Normative Landscape

The choices adolescents make about condoms and contraception are shaped by social expectations about morality, gender, and respectability. In many Kenyan and Nigerian communities, premarital sex is discouraged, discussion of sexuality with adults is limited, and maintaining a reputation for good behavior is central to family and community acceptance [11, 12]. This creates a tension between what is practiced and what can be publicly acknowledged.

Sanctions for violating these norms are swift and severe. In Kenya, pregnancy among unmarried girls is described as a source of shame that can result in ridicule or interruption of schooling [13]. In Kenya, where school re-entry policies exist, adolescents fear the social backlash and discrimination that often follow, making pregnancy the more visible and institutionally reinforced risk [14]. Girls describe injectable or oral contraception as desirable because these methods are invisible to parents, teachers, and partners [10]. This preference for covert methods is a direct response to avoiding stigma, parental and community disapproval. By contrast, condoms are public objects. Boys report discomfort buying condoms in pharmacies or shops because the act publicly marks them as sexually active and attracts moral scrutiny [7].

Health systems can add to this pressure. In both Kenya and Nigeria, adolescents describe feeling judged or questioned when seeking condoms or contraceptives, including by pharmacists and health workers [10, 15]. When services are not confidential or youth-friendly, young people turn to methods that keep their sexual activity hidden.

Table 1 summarizes this hierarchy by showing how visibility and anticipated social sanctions shape adolescents’ responses to pregnancy, condom use, and HIV risk.

**Table 1:** Normative hierarchy of adolescent risks in Kenya and Nigeria.

RISK FACTOR	CONSEQUENCE VISIBILITY	PRIMARY ANTICIPATED SANCTION	PRIMARY BEHAVIOR RESPONSE
**Pregnancy (Unintended)**	High (Physically visible, public)	Family/Community Shame, School Interruption/Expulsion, Economic Hardship	Discreet hormonal contraception (secrecy)
**Condom Use/Acquisition**	Medium (Observable in purchase setting)	Moral Judgment, Stigma of Promiscuity (especially for males)	Avoidance of purchase/dual protection, shift of responsibility to partner
**HIV Infection**	Low (Invisible until disclosure)	Stigma/Discrimination (if status known), Health decline (distant concern)	Deferral of testing, Lower prioritization of prevention compared to pregnancy
***Risk factor:*** *The outcome or behavior adolescents seek to avoid because it carries social consequences.* ***Consequence visibility:*** *How easily the outcome or behavior becomes known to others.* ***Primary anticipated sanctions:*** *The social reactions expected if norms are violated (e.g., shame or judgment).* ***Primary behavior response:*** *The actions adolescents take to manage these risks.*			

Together, these norms create a gendered pattern of protection and reinforced risk behaviors. Boys avoid condoms to protect their social reputation. Girls rely on discreet hormonal methods to prevent pregnancy. These methods do not protect against HIV or other sexually transmitted infections. In this environment, avoiding shame often matters more than avoiding infection. Programs that do not confront these social norms are likely to see continued reliance on pregnancy-only protection.

## Implications for HIV Prevention

The intertwined fears, gender roles, and moral judgments that shape adolescent behavior in Kenya and Nigeria have significant implications for HIV prevention. Programs that focus primarily on biomedical or behavioral knowledge often fail to account for the social meanings that underlie young people’s decisions. The result is a disconnect between what public health messages emphasize and what adolescents prioritize in their daily lives.

Adolescent HIV prevention programs frequently stress condom use, HIV testing, and adherence to PrEP. However, when condoms symbolize promiscuity and HIV testing implies sexual activity, both behaviors become socially risky. Studies across sub-Saharan Africa have shown that adolescents who fear social exposure or judgment are less likely to seek HIV-related services, even when they are sexually active [16, 17]. In both Kenya and Nigeria, boys’ reluctance to buy condoms and girls’ preference for discreet contraception reveal a mismatch between programmatic assumptions and the realities of adolescent social life.

This mismatch limits the effectiveness of HIV interventions. When prevention programs treat HIV risk as an individual behavioral choice, they overlook how social norms dictate which choices are even possible. For many adolescents, avoiding pregnancy safeguards their social identity, while preventing HIV protects their health. Yet health is often a distant concern compared to the immediate risk of being shamed, expelled, or disowned.

Moreover, the structural separation between SRH and HIV programs perpetuates this divide. Unmarried women who seek family planning services are often not offered HIV testing or counseling, and HIV prevention messages rarely address pregnancy-related fears. Research in Nigeria and Kenya shows that integrating HIV prevention into broader reproductive health services increases uptake and retention among adolescents, particularly when confidentiality and nonjudgmental care are ensured [18, 19]. Yet many clinics remain unprepared to offer such integration, and adolescents continue to navigate fragmented systems that force them to choose between preventing pregnancy and preventing infection.

These dynamics suggest that the fight to end HIV cannot rely on information campaigns or commodity distribution alone. Prevention must address the emotional and symbolic dimensions of adolescent decision-making. Norms of shame, secrecy, and respectability operate as invisible barriers that undermine technical interventions. Until HIV prevention strategies engage directly with these social forces, the most affected group, adolescent girls and young women, and adolescent boys and young men, will remain caught between managing visibility and managing risk.

In practice, this means reframing prevention not as a message about disease but as a conversation about dignity, choice, and trust. Programs stand a better chance of succeeding when condom use is framed as a sign of responsibility and self-protection, rather than evidence of promiscuity. Creating space for open, age-appropriate conversations about sexual and reproductive health and disease prevention in schools and within families and engaging both boys and girls in redefining what “being protected” means can help shift these perceptions. The core challenge is not only increasing access to HIV services, but ensuring these services are viewed as acceptable and aligned with the moral and social realities young people navigate.

## Conclusion: Reframing HIV Programming with Norm-Sensitive Interventions

The reflections from Kenya and Nigeria reveal that HIV prevention among adolescents cannot be understood without examining the moral and social context in which decisions are made. The fear of pregnancy, the silence surrounding sexuality, and the moral weight of respectability all influence how young people perceive and manage risk. For many adolescents, pregnancy represents public shame, while HIV is seen as a private misfortune. This hierarchy of fear, shaped by social norms and reinforced by gender expectations, continues to undermine the effectiveness of prevention strategies. Hence, a norm-sensitive approach requires programs to focus on how adolescents interpret protection within their social environment. In both countries, prevention choices are shaped less by lack of knowledge and more by how adults and peers judge visible behaviors. The GNLA study found that even when adolescents understand the value of condoms or HIV testing, they often avoid them to prevent public criticism from friends, partners, parents, or community members [5].

The global HIV response must move beyond technical solutions, transforming how prevention is imagined and delivered by engaging with the social realities that define adolescents’ choices, and this analysis calls for action among global HIV program designers and implementers to respond to these realities in how prevention strategies are designed and delivered. Such action must center the voices of adolescents, especially girls, who live at the intersection of health vulnerability and social control. It also requires re-educating the adults who shape and enforce norms, from parents and teachers to health providers and religious leaders. When conversations about sexuality shift from judgment to empathy, and when protection becomes a shared expression of responsibility, the barriers created by fear and shame begin to fade away.

Success will depend on our ability to redefine what it means to be responsible, respectable, and safe. When young people no longer have to choose between social acceptance and health protection, the path toward an HIV-free generation will become possible.
